# Prevalence of Asthma Among Adults in Metropolitan Versus Nonmetropolitan Areas in Montana, 2008

**Published:** 2011-12-15

**Authors:** Jessie C. Frazier, Todd S. Harwell, Katie M. Loveland, Heather J. Zimmerman, Steven D. Helgerson

**Affiliations:** Asthma Control Program, Montana Department of Public Health and Human Services, Helena, Montana; Montana Department of Public Health and Human Services; Asthma Control Program, Montana Department of Public Health and Human Services, Helena, Montana; Asthma Control Program, Montana Department of Public Health and Human Services, Helena, Montana; Asthma Control Program, Montana Department of Public Health and Human Services, Helena, Montana

## Abstract

**Introduction:**

Most US studies on asthma prevalence have been conducted in urban areas, and few have assessed the prevalence of asthma among residents of rural areas versus urban areas. The objective of this study was to compare the prevalence of asthma among adults living in metropolitan versus nonmetropolitan counties in Montana.

**Methods:**

We analyzed data from 6,846 adult Montanans who completed the Behavioral Risk Factor Surveillance System survey in 2008. We used Rural-Urban Continuum Codes to categorize respondents' county of residence as metropolitan (Metro), nonmetropolitan and adjacent to a metropolitan county (NMA), and nonmetropolitan and nonadjacent to a metropolitan county (NMNA). We compared the prevalence of current self-reported asthma among respondents in the 3 areas, overall and by selected characteristics, and conducted multivariable logistic regression analyses to identify factors independently associated with current self-reported asthma.

**Results:**

No differences in the prevalence of self-reported asthma were found between residents of Metro and NMA or NMNA counties, overall or by age, sex, race, years of education, health insurance status, annual household income, or body mass index. Respondents aged 65 years or older (adjusted odds ratio [AOR], 0.7; 95% confidence interval [CI], 0.5-0.9) and men (AOR, 0.6; 95% CI, 0.5-0.8) were less likely to report current asthma than younger respondents and women, respectively. Obese respondents were more likely (AOR, 1.9; 95% CI, 1.4-2.7) to report asthma than were respondents who were not obese. Metropolitan county of residence was not independently associated with self-reported current asthma.

**Conclusion:**

The prevalence of self-reported current asthma is similar in metropolitan and nonmetropolitan counties in Montana, but other sociodemographic differences exist. Our findings highlight the need to conduct regional and state surveillance of asthma to understand the demographic risk factors associated with it and to determine the potential geographic variation of asthma prevalence in the United States.

## Introduction

Asthma is one of the most common chronic illnesses in the United States and is a public health concern because of its health care–related costs and morbidity (1). In 2008, asthma prevalence in the United States was 8.5% and in Montana was 9.6%, representing an estimated 70,500 people in the state. (2). Previous US studies suggest that asthma prevalence is higher among subgroups of the population and that it varies geographically (1-5). Geographic differences in asthma prevalence may be associated with differences in sociodemographic characteristics and other risk factors. Most published studies in the United States on asthma prevalence have been conducted in urban areas (6), and few state or national studies have been conducted to compare the prevalence of asthma among people living in urban areas with that of people living in rural areas (6-10).

Some European studies have found that people born and raised in rural settings, particularly farming communities, may be at decreased risk of developing asthma (11-13). The "hygiene hypothesis" suggests that environmental exposures early in life influence immunologic protection against the development of asthma (eg, environmental exposure in farming communities) (14). Both the lack of epidemiologic studies in rural areas and the hygiene hypothesis may influence the belief that asthma is less common among people living in rural areas.

Montana is the fourth-largest US state in area and one of the least densely populated states. According to 2010 census data, Montana's population was 989,415 and its population density was 6.8 people per square mile (compared with 87.4 people per square mile nationally) (15-16). A larger proportion of Montanans are non-Hispanic white (91%) or American Indian/Alaska Native (6%) compared with the national population (75% non-Hispanic white, 1% American Indian/Alaska Native) (15). Compared with the national population, Montanans are older (mean age, 37.5 y vs 35.3 y) and have a lower annual household income ($33,024 vs $41,994).

In 2008, the Montana Department of Public Health and Human Services (DPHHS) conducted the Behavioral Risk Factor Surveillance System (BRFSS) survey, a random-digit–dialed telephone survey of a representative sample of noninstitutionalized adults (17), to assess the prevalence of self-reported asthma among adults in the state. The Montana DPHHS has conducted the BRFSS annually since 1984, and 6,846 Montanans responded to the survey in 2008 (48.3% response rate vs 53.3% nationally). The objective of this study was to compare the prevalence of asthma among adults living in metropolitan versus nonmetropolitan counties in Montana.

## Methods

The BRFSS survey includes questions about sociodemographic characteristics, including age, sex, race, annual household income, years of education, and health insurance status. The survey also asks respondents to report their height and weight, which are used to calculate body mass index (BMI, kg/m^2^). Each respondent's BMI was categorized as overweight (25.0-29.9), obese (≥30.0), or not overweight or obese (<25.0). The survey asks, "Have you ever been told by a doctor, nurse, or other health professional that you had asthma?" Respondents indicating yes to that question were then asked "Do you still have asthma?" Respondents indicating yes to this second question were considered to currently have asthma.

BRFSS uses disproportionate, stratified sampling. We reweighted the 2008 data to be representative of the adult Montana population by using the Office of Management and Budget's Rural-Urban Continuum Codes (RUCC) (18). The RUCC groups counties into 1 of 9 categories on the basis of their overall population size for metropolitan areas or on the basis of their degree of urbanization and adjacency to a metropolitan area for nonmetropolitan counties. In 2000, Montana had 4 counties that were classified as a metropolitan area with a population of fewer than 250,000. Of nonmetropolitan counties not adjacent to a metropolitan area, 4 had a population of 20,000 or more, 15 had a population of 2,500 to 19,999, and 20 had fewer than 2,500 residents. Of nonmetropolitan counties adjacent to a metropolitan area, 3 had a population of 2,500 to 19,999, and 10 had a population of fewer than 2,500 residents. We combined these subgroups of counties into 3 categories for analyses: the 4 counties in metropolitan areas (Metro) (n = 1,407), the 13 nonmetropolitan counties adjacent to a metropolitan county (NMA) (n = 1,262), and the 39 nonmetropolitan counties nonadjacent to a metropolitan county (NMNA) (n = 4,157).

We conducted data analyses using SAS version 9.2 complex survey procedures (SAS Institute, Inc, Cary, North Carolina). We calculated weighted prevalence estimates and 95% confidence intervals (CIs) to compare the sociodemographic characteristics of respondents from the 3 areas. We also calculated weighted prevalence estimates, 95% CIs, and odds ratios (ORs) to compare the prevalence of current self-reported asthma among respondents in the 3 areas, overall and by selected characteristics. We conducted multivariable logistic regression analyses to identify factors independently associated with current self-reported asthma. Significance was set at *P* ≤ .05.

## Results

Respondents in NMA counties were older than respondents in Metro counties (Table 1). Compared with Metro and NMNA counties, NMA counties had a larger proportion of nonwhite respondents, lower annual household income, a smaller proportion of respondents with more than 12 years of education, and a larger proportion of respondents without health insurance.

The prevalence of asthma was 9.9% among respondents living in a Metro county, 10.2% among respondents living in an NMA county, and 9.3% for respondents living in an NMNA county. We found no differences between residents of Metro and NMA or NMNA counties in the prevalence of self-reported asthma, overall or by age, sex, race, annual household income, years of education, health insurance status, or BMI (Table 2).

Respondents aged 65 years or older (compared with those aged 18-44) and men (compared with women) were less likely to have current asthma (Table 3). Respondents who were obese were more likely to report current asthma than were respondents who were not overweight or obese. Respondents from NMA and NMNA counties were not more likely to report current asthma than were respondents from Metro counties.

## Discussion

Our findings suggest that there are no differences in the prevalence of self-reported asthma among adults in metropolitan and nonmetropolitan (or rural) areas in Montana. According to our analyses, only being younger, female, or obese was associated with a higher prevalence of self-reported asthma. Metropolitan county of residence was not independently associated with current self-reported asthma.

A 2009 study by Morrison et al, also using BRFSS data, found that the prevalence of self-reported asthma was similar in urban and rural counties in the United States (7). Prevalence rates of current asthma in our study ranged narrowly from 7.8% in metropolitan areas to 7.9% in rural areas. Morrison et al found that other demographic and health-related factors — including being younger, female, nonwhite, poorly educated, and overweight or obese, and having a low annual household income and no health insurance — were associated with asthma. A cross-sectional study in Canada found similar prevalence rates of asthma among urban (7.7%; 95% CI, 7.1-8.3) and rural (6.7%; 95% CI, 5.7-7.7) adults in 2000 to 2001 (19).

Our study has limitations. First, the Montana BRFSS response rate was low (48%). However, self-reported current asthma prevalence estimates from other national studies with higher response rates (69%) are similar to ours (20). Second, the BRFSS does not include institutionalized adults or adults in households without landline telephones. However, in 2000, only approximately 3% of the Montana population did not have access to a landline telephone (21). Calling cellular telephones to select potential respondents is being piloted by the Montana BRFSS, but no responses from participants using a cellular telephone were included in the final data set in 2008. Young people, who are more likely to have only a cellular telephone in their home, were likely to be underrepresented in 2008. However, approximately 25% of respondents from Metro counties were in the younger age group; the percentage was similar for the NMA and NMNA respondents. Third, the prevalence of current asthma was based on self-report, which may not reflect the true prevalence of diagnosed asthma. Previous studies that compared self-reported asthma status with a clinical diagnosis, however, suggest that self-reported asthma is accurate and reliable (22). Fourth, the 4 Montana counties categorized as Metro are large geographically and have rural-associated industries, including agriculture. According to 2000 census data, however, less than 4% of the employed population in those counties worked in the agricultural industry (15). Finally, our findings are limited to Montana and may not be generalizable to other communities or regions in the United States.

Our findings suggest that there is no disparity in the prevalence of asthma between adults living in metropolitan versus nonmetropolitan areas in Montana. In both areas, the prevalence of asthma is high. Our findings highlight the need to conduct regional and state surveillance of asthma to understand the demographic risk factors associated with it and to determine the potential geographic variation of asthma prevalence in the United States. Surveillance to assess the level of control across these geographic subpopulations is also needed. The Centers for Disease Control and Prevention, in collaboration with US states, has implemented the Asthma Call-back Survey, which may be used to provide this information (23).

## Figures and Tables

**Table 1:** Demographic Characteristics of Survey Respondents (n = 6,846), by Geographic Designation, Behavioral Risk Factor Surveillance System, Montana, 2008

Characteristic	County Designation			*P* Value^d^

	Metropolitan^a^ (n = 1,411), % (95% CI)	Nonmetropolitan Adjacent^b^ (n = 1,267), % (95% CI)	Nonmetropolitan Nonadjacent^c^ (n = 4,168), % (95% CI)	
**Age, y**				
18-44	47.8 (44.2-51.3)	39.9 (35.5-44.4)	43.9 (41.7-46.2)	.01
45-64	35.7 (32.6-38.7)	38.5 (34.8-42.2)	37.8 (35.9-39.7)	
≥65	16.6 (14.8-18.3)	21.6 (19.0-24.2)	18.3 (17.0-19.5)	
**Sex**				
Male	49.2 (45.7-52.7)	49.8 (45.7-53.9)	49.4 (47.2-51.6)	.99
Female	50.8 (47.3-54.3)	50.2 (46.1-54.3)	50.6 (48.4-52.8)	
**Race**				
White	95.2 (93.5-96.9)	85.8 (82.1-89.4)	93.4 (92.3-94.4)	<.001
Other	4.8 (3.1-6.5)	14.2 (10.6-17.9)	6.6 (5.4-7.7)	
**Annual household income, $**				
<25,000	19.2 (16.5-22.0)	27.6 (24.1-31.1)	21.3 (19.6-23.1)	<.001
25,000-49,999	28.6 (25.4-31.7)	28.7 (24.7-32.6)	29.3 (27.3-31.3)	
≥50,000	42.2 (38.8-45.6)	31.4 (27.7-35.2)	37.8 (35.7-39.9)	
Unknown	10.0 (8.1-11.9)	12.3 (9.8-14.8)	11.6 (10.2-13.0)	
**Education, y**				
<12	4.9 (3.5-6.2)	7.7 (5.6-9.8)	6.8 (5.6-7.9)	<.001
12	27.9 (24.8-31.1)	36.2 (32.0-40.4)	31.4 (29.3-33.4)	
>12	67.2 (63.9-70.5)	56.1 (51.9-60.3)	61.9 (59.7-64.0)	
**Health insurance status**				
Has insurance	87.1 (84.6-89.6)	77.1 (73.2-80.9)	82.6 (80.8-84.4)	<.001
Has no insurance	12.9 (10.4-15.4)	22.9 (19.1-26.7)	17.4 (15.6-19.2)	
**Body mass index, kg/m^2^**				
<25.0 (not overweight or obese)	36.1 (32.7-39.5)	39.5 (35.2-43.7)	39.1 (36.9-41.2)	.51
25.0-29.9 (overweight)	39.6 (36.1-43.1)	36.3 (32.4-40.2)	37.1 (35.0-39.2)	
≥30.0 (obese)	24.3 (21.3-27.3)	24.3 (20.8-27.7)	23.8 (22.0-25.7)	

Abbreviation: CI, confidence interval.

a Counties in metropolitan areas of fewer than 250,000 people.

b Nonmetropolitan counties adjacent to a metropolitan area.

c Nonmetropolitan counties not adjacent to a metropolitan area.

d *P* values calculated by using χ^2^ test.

**Table 2:** Current Asthma Prevalence Among Survey Respondents (n = 6,846), Overall and by Selected Characteristics, by Geographic Designation, Behavioral Risk Factor Surveillance System, Montana, 2008

Characteristic	County Designation				
	Metropolitan^a^ (n = 1,411), % (95% CI)	NMA^b^ (n = 1,267)		NMNA^c^ (n = 4,168)	
		% (95% CI)	OR (95% CI)^d^	% (95% CI)	OR (95% CI)^e^
**Total**	9.9 (7.9-11.9)	10.2 (7.8-12.7)	1.0 (0.7-1.5)	9.3 (8.0-10.6)	0.9 (0.7-1.2)
**Age, y**					
18-44	9.7 (6.1-13.2)	12.7 (7.3-18.0)	1.4 (0.7-2.6)	9.8 (7.3-12.2)	1.0 (0.6-1.7)
45-64	11.1 (8.3-13.8)	10.0 (7.1-13.0)	0.9 (0.6-1.4)	9.5 (7.9-11.1)	0.8 (0.6-1.2)
≥65	8.2 (5.5-10.9)	6.1 (3.7-8.5)	0.7 (0.4-1.3)	7.6 (5.9-9.3)	0.9 (0.6-1.4)
**Sex**					
Male	6.7 (4.1-9.2)	7.9 (4.4-11.3)	1.2 (0.6-2.2)	8.3 (6.4-10.2)	1.3 (0.8-2.0)
Female	13.0 (10.0-16.0)	12.6 (9.1-16.1)	1.0 (0.6-1.5)	10.2 (8.5-11.9)	0.8 (0.5-1.1)
**Race**					
White	9.9 (7.9-12.0)	8.8 (6.5-11.2)	0.9 (0.6-1.3)	9.1 (7.8-10.4)	0.9 (0.7-1.2)
Other	11.9 (1.1-22.8)	18.8 (8.4-29.2)	1.7 (0.5-6.0)	11.9 (6.1-17.6)	1.0 (0.3-3.2)
**Annual household income, $**					
<25,000	13.9 (9.0-18.8)	15.3 (9.5-21.0)	1.1 (0.6-2.0)	10.2 (7.7-12.7)	0.7 (0.4-1.1)
25,999-49,999	10.0 (6.2-13.8)	10.0 (4.9-15.2)	1.0 (0.5-2.0)	7.8 (5.8-9.9)	0.8 (0.5-1.3)
≥50,000	8.0 (5.1-11.0)	7.7 (4.3-11.0)	0.9 (0.5-1.8)	10.2 (7.9-12.5)	1.3 (0.8-2.1)
Unknown	10.3 (3.6-17.0)	6.1 (2.3-9.8)	0.6 (0.2-1.5)	8.4 (4.4-12.4)	0.8 (0.3-1.9)
**Education, y**					
<12	7.3 (0-14.8)	11.6 (4.4-18.7)	1.7 (0.4-6.1)	6.9 (3.0-10.7)	0.9 (0.3-3.3)
12	12.8 (8.3-17.3)	9.8 (5.5-14.1)	0.7 (0.4-1.4)	10.1 (7.6-12.7)	0.8 (0.5-1.3)
>12	8.9 (6.6-11.1)	10.3 (7.0-13.7)	1.2 (0.8-1.9)	9.1 (7.6-10.7)	1.0 (0.7-1.4)
**Health insurance status**					
Has insurance	10.0 (7.9-12.1)	11.7 (8.7-14.7)	1.2 (0.8-1.7)	9.2 (7.8-10.5)	0.9 (0.7-1.2)
Has no insurance	9.5 (3.4-15.6)	6.3 (2.4-10.3)	0.6 (0.2-1.7)	9.9 (6.5-13.3)	1.0 (0.5-2.3)
**Body mass index, kg/m^2^**					
<25.0 (not overweight or obese)	7.2 (4.4-10.0)	7.5 (3.7-11.3)	1.0 (0.5-2.1)	7.9 (5.8-10.0)	1.1 (0.7-1.9)
25.0-29.9 (overweight)	8.4 (5.2-11.6)	9.9 (6.4-13.4)	1.2 (0.7-2.1)	8.8 (5.8-10.0)	1.1 (0.6-1.7)
≥30.0 (obese)	15.3 (10.6-20.0)	15.0 (8.7-21.4)	1.0 (0.5-1.8)	11.9 (9.0-14.7)	0.7 (0.5-1.2)

Abbreviations: NMA, nonmetropolitan adjacent; NMNA, nonmetropolitan nonadjacent; OR, odds ratio; CI, confidence interval.

a Counties in metropolitan areas of fewer than 250,000 people.

b Nonmetropolitan counties adjacent to a metropolitan area.

c Nonmetropolitan counties not adjacent to a metropolitan area.

d Comparison between NMA counties and metropolitan counties.

e Comparison between NMNA counties and metropolitan counties.

**Table 3:** Factors Independently Associated With Self-Reported Current Asthma Among Survey Respondents (n = 6,846), Behavioral Risk Factor Surveillance System, Montana, 2008

**Factor**	ß (SE)	AOR (95% CI)^a^
**Age, y**		
18-44	1 [Reference]	
45-64	0.110 (0.075)	1.0 (0.7-1.3)
≥65	−0.260 (0.089)	0.7 (0.5-0.9)
**Sex**		
Female	1 [Reference]	
Male	−0.219 (0.066)	0.6 (0.5-0.8)
**Race**		
White	−0.224 (0.119)	0.6 (0.4-1.0)
Other	1 [Reference]	
**Annual household income, $**		
<25,000	0.242 (0.109)	1.3 (1.0-1.8)
25,999-49,999	−0.079 (0.111)	1.0 (0.7-1.3)
≥50,000	1 [Reference]	
Unknown	−0.118 (0.167)	0.9 (0.6-1.5)
**Education, y**		
<12	−0.228 (0.173)	0.8 (0.4-1.3)
12	0.171 (0.113)	1.1 (0.9-1.5)
>12	1 [Reference]	
**Health insurance status**		
Has insurance	1 [Reference]	
Has no insurance	−0.179 (0.010)	0.7 (0.5-1.0)
**Body mass index, kg/m^2^**		
<25.0 (not overweight or obese)	1 [Reference]	
25.0-29.9 (overweight)	−0.053 (0.086)	1.3 (0.9-1.8)
≥30.0 (obese)	0.360 (0.086)	1.9 (1.4-2.7)
**Geographic designation**		
Metropolitan^b^	1 [Reference]	
Nonmetropolitan adjacent^c^	0.042 (0.104)	1.0 (0.7-1.5)
Nonmetropolitan nonadjacent^d^	−0.041(0.080)	1.0 (0.7-1.3)

Abbreviations: SE, standard error; AOR, adjusted odds ratio; CI, confidence interval.

a Each of the variables in the table was included in the model.

b Counties in metropolitan areas of fewer than 250,000 people.

c Nonmetropolitan counties adjacent to a metropolitan area.

d Nonmetropolitan counties not adjacent to a metropolitan area.
